# Multinational development of a questionnaire assessing patient satisfaction with anticoagulant treatment: the 'Perception of Anticoagulant Treatment Questionnaire' (PACT-Q^©^)

**DOI:** 10.1186/1477-7525-7-9

**Published:** 2009-02-06

**Authors:** Martin H Prins, Alexia Marrel, Paulo Carita, David Anderson, Marie-Germaine Bousser, Harry Crijns, Silla Consoli, Benoit Arnould

**Affiliations:** 1 Department of Epidemiology, Care and Public Health Research Institutes, University of Maastricht, the Netherlands; 2 Department of Clinical Epidemiology and Medical Technology Assessment, Academic Hospital, Maastricht, the Netherlands; 3 Mapi Values, Lyon, France; 4 Sanofi-Aventis, Paris, France; 5 Dalhousie University, Halifax, Nova Scotia, Canada; 6 Hôpital Lariboisière, Paris, France; 7 Hôpital Européen Georges-Pompidou, Paris, France

## Abstract

**Background:**

The side effects and burden of anticoagulant treatments may contribute to poor compliance and consequently to treatment failure. A specific questionnaire is necessary to assess patients' needs and their perceptions of anticoagulant treatment.

**Methods:**

A conceptual model of expectation and satisfaction with anticoagulant treatment was designed by an advisory board and used to guide patient (n = 31) and clinician (n = 17) interviews in French, US English and Dutch. Patients had either atrial fibrillation (AF), deep venous thrombosis (DVT), or pulmonary embolism (PE). Following interviews, three PACT-Q language versions were developed simultaneously and further pilot-tested by 19 patients. Linguistic validations were performed for additional language versions.

**Results:**

Initial concepts were developed to cover three areas of interest: 'Treatment', 'Disease and Complications' and 'Information about disease and anticoagulant treatment'. After clinician and patient interviews, concepts were further refined into four domains and 17 concepts; test versions of the PACT-Q were then created simultaneously in three languages, each containing 27 items grouped into four domains: "Treatment Expectations" (7 items), "Convenience" (11 items), "Burden of Disease and Treatment" (2 items) and "Anticoagulant Treatment Satisfaction" (7 items). No item was deleted or added after pilot testing as patients found the PACT-Q easy to understand and appropriate in length in all languages. The PACT-Q was divided into two parts: the first part to measure the expectations and the second to measure the convenience, burden and treatment satisfaction, for evaluation prior to and after anticoagulant treatment, respectively. Eleven additional language versions were linguistically validated.

**Conclusion:**

The PACT-Q has been rigorously developed and linguistically validated. It is available in 14 languages for use with thromboembolic patients, including AF, PE and DVT patients. Its validation and psychometric properties have been tested and are presented in a separate manuscript.

## Background

Thromboembolic events are a major cause of mortality and morbidity in Western societies [1-3]. Such events occur when a mechanical mass, termed thrombus, obstructs vascular blood flow locally or detaches and clots to occlude blood flow downstream. Thromboembolic events or recurrences thereof can be effectively reduced by the use of anticoagulants. Currently, three conditions constitute the majority of indications for long-term anticoagulant treatment: atrial fibrillation (AF), where anticoagulants are used to prevent stroke, and deep venous thrombosis (DVT) and pulmonary embolism (PE), where anticoagulants are used to prevent recurrent disease.

Standard long-term anticoagulant treatment consists of oral vitamin K antagonists (VKA) including warfarin, phenprocoumon and acenocoumarol. However, there are well-known drawbacks in the routine medical use of VKA. For example, all must be given daily and have interactions with food and a many commonly used drugs, which is a problem since many patients requiring anticoagulant therapy are elderly. Moreover, VKA potencies vary between patients, resulting in unpredictable pharmacodynamic effects and requiring regular monitoring. Significant side effects can also occur, which are most prominently bleeding disorders.

As a result, these drawbacks are likely to impose a significant burden on patients (e.g. complicated and frequent monitoring, side effects) and probably affect their health-related quality of life (HRQoL). In fact, most patients eligible to receive VKA do not receive optimal treatment. Instead, they receive less effective doses or no therapy at all [4-9].

Intense research is currently underway in an effort to develop safer and more effective anticoagulants. Some of these have the advantage of an increased half-life, allowing for once-a-week administration [10]. Others have the potential to be given orally, without laboratory monitoring [11,12]. For assessing the real value of new drugs in this field, the evaluation of patients' perspectives and satisfaction towards these treatments will be necessary. Traditional efficacy endpoints alone may not be able to include all the benefits of novel therapies such as the reduction in treatment burden.

With the help of patient-reported outcome (PRO) questionnaires, including treatment satisfaction questionnaires, treatment benefits for the patient are now often evaluated in clinical trials [13,14]. Treatment satisfaction is a concept that is distinct from other PROs as it focuses on the patients' rating of salient aspects of a treatment experience. These ratings are determined by comparisons with the patients' subjective standards, formed by expectations, past experiences, personality characteristics, values and beliefs [15-17]. Failure to achieve sufficient treatment satisfaction has been reported to cause poor treatment compliance [18-20], which in turn may diminish the effectiveness of treatments – especially among patients with chronic conditions [21,22].

In order to evaluate the full benefits of anticoagulant treatments, a specific patient-reported questionnaire that assesses patients' satisfaction with anticoagulant treatment is thus required. Ideally, this questionnaire must be applicable to a wide range of patients receiving anticoagulant therapies and must also address issues related to treatment attributes such as the route of administration (e.g. subcutaneous *versus *oral). In addition, this questionnaire should achieve currently recommended validation standards and be available in several languages for use in multinational clinical trials.

A literature search led to the conclusion that no questionnaire meeting all these requirements exists for evaluating anticoagulant treatments [7,23-30]. Therefore, we developed and validated a patient-reported treatment satisfaction questionnaire, the 'Perception of Anticoagulant Treatment Questionnaire' (PACT-Q), in several languages, using a wide spectrum of thromboembolic patients and following currently recommended methodology.

## Methods

### Participants

#### Advisory Board

An advisory board consisting of 8 experts from a diverse range of disciplines, was set up to provide expert input on all stages of development for the questionnaire and to work with a team of questionnaire specialists. Their input consisted in creating a conceptual model, making choices to optimize the questionnaire development process, validating the results at each critical step of development and providing final decisions on subsequent procedures.

#### Clinician and patient recruitment

Clinicians were recruited among specialists in DVT, PE and/or AF, from France, the Netherlands and the US.

Patients were recruited in France, the Netherlands and the US, either by the specialists who were interviewed or by specialists from our network. In order to complete the targeted sample, three patients were recruited via an advertising campaign on a thrombosis website in the Netherlands, and five were recruited via an agency specialised in patient recruitment in the US. They had to be over 18 years-old, have had a thromboembolic event within the last two years, or AF for at least three months prior to the interview. Patients also had to have taken an anticoagulant within the three months prior to the interview, be willing and able to participate in a one-hour interview and speak the local language fluently. They were asked to provide a written consent regarding their participation in the study. Patients with psychotic or psychiatric diseases, newly diagnosed serious chronic conditions other than AF, or a rating of 4 or 5 on the Rankin Scale were excluded from the study. To ensure a broad spectrum of patients, the population was to include one AF patient who was still at work per country, half of all DVT/PE patients had to be aged 50 or below, and patients had to have different levels of education for all disease conditions.

### Concept development

The advisory board first met to generate an initial list of concepts related to the expectations and satisfaction of patients with anticoagulant treatment. The concept list was created in English and was based on the patients' main concerns found in a literature review and subsequently completed with the collective experience of the individual advisory board members. This concept list provided the structure for designing the clinician interview guide.

The objectives of the guide were to 1) capture clinicians' personal experience in the fields of DVT, PE and/or AF, 2) collect their opinions on the current state of disease management and treatments, 3) document the improvements that were needed in the treatment and management of these disorders and 4) discuss their impressions of the patients' experience and concerns regarding their disorders and treatments. The final guide was developed in UK English, then validated by the advisory board and translated into US English, Dutch and French.

Concepts can be general or highly specific; in the text, we use the term "detailed concepts" at the more specific level, which can be assessed with a specific item in the questionnaire; detailed concepts that are closely related are grouped in "concepts", and concepts that are expected to be pooled to calculate a score are further grouped in a "domain".

#### Clinician interviews

Trained researchers from the native language of the interviewees conducted all interviews. Interviews were performed to amend and complete a list of concepts defined during the first advisory board meeting and to enable the writing of a guide for patient interviews. Clinician interviews were conducted over the phone, recorded and transcribed into grids. Transcripts were analysed in each country and were used to amend and complete the initial list of concepts. Concepts within this second list were categorised into new global concepts and detailed concepts sections. Results from each country were consolidated to create an international list.

From this list, a patient guide was designed for the patient interviews. The objectives of the guide were to 1) collect patients' opinions and perspectives on the management and treatment of their disease in their own words, 2) identify the important aspects of their treatment and disease management, 3) identify how patients assess the efficacy and safety of their anticoagulant treatment and their preferences, 4) identify advantages and constraints related to anticoagulant treatment as perceived by patients and 5) identify patients' worries and expectations concerning anticoagulant treatment and medical follow-up. The final guide, developed in UK English, was also validated by the advisory board and translated into US English, Dutch and French.

#### Patient interviews

Patient interviews were performed to test the list of concepts and to collect patient responses in their own wording to create the items of the questionnaire. A target goal of 30 patient interviews (ten per country) was set prior to recruiting patients. In each country, three patients with DVT, three patients with PE and four patients with AF were to be recruited to provide a relevant spectrum of patients. All the research processes were conducted following the tenets of the Declaration of Helsinki.

Interviews were recorded and transcribed into a grid. Verbatim transcripts were analysed by clinical condition, in each language, and used to amend and complete the international list of concepts. All global and detailed concepts were then translated into English and used to create the questionnaire items.

### Item generation

Items were generated simultaneously in Dutch, French and US English during a three-day 'item generation meeting' with questionnaire specialists. Briefly, relevant verbatim responses were first selected from patient transcripts, analysed and organised into a list of concepts then further grouped into domains. A short list of detailed concepts was selected. Following review and validation of the short list by the advisory board, items were drafted to provide the first version of the questionnaire. The advisory board then validated the first US English, Dutch and French version of the questionnaire.

### Content validity testing

Native speaking interviewers conducted content validity interviews. The goal was to assess the ease of comprehension, clarity, cultural equivalence, preference and appropriateness of the first version of the PACT-Q (instructions, questionnaire items and response scales). Interviews were performed with patients other than those who participated in the concept development phase, but recruited following the same criteria; one extra criterion was added, consisting in the inclusion of one DVT and one PE patient currently receiving or having received subcutaneous injection of anticoagulant treatment during the two months prior to the interview.

A patient interview guide was developed in UK English and translated into Dutch, US English and French. Interviews with patients with prior DVT, PE and AF were performed face-to-face at home, over the phone, or at hospital and transcribed into grids. Relevant comments were all translated into English. If required, they were reformulated in both English and the target language to make them clearer and easier to understand. The pilot version of the questionnaire was then produced and validated by the advisory board.

### Linguistic validation

An internationally acknowledged translation methodology was used in order to obtain eleven additional language versions that were conceptually equivalent and easily understandable by each of the target population [31,32]. For seven of the languages (Czech, Danish, Canadian French, German, Italian, Polish and US Spanish), translation followed a standard linguistic validation process, which included a conceptual analysis of the original instrument, the recruitment and briefing of a consultant in each target country, a forward translation step, a backward translation step, a pilot-testing step (clinician reviewing and cognitive debriefing with five patients in each target country) and two final proof-readings (one by clinicians and one by patients). For the four remaining languages (Belgian Dutch, Australian English, Canadian English, and Belgian French), that were closely similar to previously validated language versions (e.g. Dutch/Belgian Dutch), an adjusted validation process was performed. The adjusted validation included all the standard validation procedures with the exception of the forward and backward translations that were replaced by a language adaptation step.

## Results

### Participant characteristics

#### Description of the clinicians

A total of 17 clinicians were interviewed: France (n = 6), the Netherlands (n = 6) and the US (n = 5). Clinicians were cardiologists, internists, respirologists and vascular medicine specialists, with extensive experience (from 4 to 37 years) with DVT, PE and AF patients. The mean number of DVT and PE patients treated per year by clinicians was 189 and the mean number of AF patients treated per year was 129. Interviews were one-hour long on average.

#### Description of the patients

The socio-demographic characteristics of the interviewed patients are provided according to disease condition in Tables 1 and 2. Thirty-one patients, 14 males and 17 females, were interviewed for all three countries. Nine patients were diagnosed with DVT, 9 with PE and 13 with AF (Table 2). The interviewed population was heterogeneous in terms of age, employment status, treatment experience and perspective towards anticoagulant treatment. For example, among the 31 patients interviewed, eleven were actively working or homemakers, 16 were retired, one was unemployed, one was disabled and two were unable to work due to their health status (Table 1). The mean patient age was 57 years; on average AF patients were older than patients with DVT or PE (69 *versus *47 and 51 years respectively). Anticoagulant treatment experience was also longer in AF patients than in DVT and PE patients (respective mean duration of 5.6, 1.2 and 2.3 years).

**Table 1:** Patient socio-demographic characteristics according to disease condition

	**DVT** **(n = 9)**	**PE** **(n = 9)**	**AF** **(n = 13)**	**Total** **(n = 31)**
**COUNTRY (n)**				
France	3	3	5	11
The Netherlands	3	3	4	10
The United States	3	3	4	10
				
**GENDER (n)**				
Male	5	2	7	14
Female	4	7	6	17
				
**AGE (years)**				
Range	21 – 77	30 – 74	39 – 79	21 – 79
Mean	47.4	51.3	68.5	57.4
				
**LIVING SITUATION (n)**				
Living alone	3	.	5	8
Living as a couple	6	9	8	23
				
**LEVEL OF EDUCATION (n)**				
Primary school	1	.	3	4
High school diploma	3	3	3	9
Some college or vocational school	1	3	4	8
College or university degree	3	2	1	6
Graduate or professional school	1	1	1	3
Other, please specify	.	.	1 (grammar school)	1
				
**EMPLOYMENT STATUS (n)**				
Full-time paid employment	4	1	1	6
Part-time paid employment	1*	1*	1	3
Homemaker/housewife	.	1	1	2
Retired	3	3	10	16
Unemployed	.	1	.	1
Not working due to present health status	.	2	.	2
Other, please specify	1 (disabled)	.	.	1

* part-time because partly unable to work due to health status

AF, atrial fibrillation; DVT, deep venous thrombosis; PE, pulmonary embolism

**Table 2:** Initial interviews: patient clinical characteristics according to disease condition

	**DVT** **(n = 9)**	**PE** **(n = 9)**	**AF** **(n = 13)**	**Total** **(n = 31)**
**DISEASE INFORMATION: Duration of AF or last PE/DVT (years)**				
Range	0.3 – 2.6	0.6 – 3	0.4 – 13	0.3 – 13
Mean	1.3	1.2	5.8	3.1
**TREATMENT INFORMATION**				
**Name(s) of anticoagulation treatment(s)**				
Previscan	1	1	3	5
Coumadine	5	5	6	16
Fenprocoumon	.	.	1	1
Fraxodi	.	1	.	1
Sintrom	.	.	1	1
Marcoumar	2	1	2	5
Acenocoumarol	1	1	.	2
**Current anticoagulant administration route**				
Oral	9	8	13	30
Injection	.	1	.	1
**Experience of anticoagulant self-injections**				
Yes	3	3	2	8
No	6	6	11	23
**Blood test frequency**				
Every 1 – 2 weeks	5	5	3	13
Every 2 – 4 weeks	2	2	3	7
Every month	2	2	4	8
Every 1–2 months	.	.	3	3
**Receiving an anticoagulant treatment since (year)**				
Range	0.3 – 2	0.6 – 8	0.5 – 13	0.3 – 13
Mean	1.2	2.3	5.6	3.3
**Duration of anticoagulant treatment**				
Lifetime	2	5	12	19
One year	.	2	.	2
6 months	3	1	1	5
Less than 6 months	1	1	.	2
Unknown	2	.	.	2
Finished	1	.	.	1
**OTHER CO-MORBIDITIES**				
Yes	4	5	8	17
No	5	4	5	14

AF, atrial fibrillation; DVT, deep venous thrombosis; PE, pulmonary embolism

### Concept development

Based on the advisory board deliberation, concepts were initially grouped into three areas of interest: 1) Treatment, 2) Disease and Complications and 3) Information about disease and anticoagulant treatment. After clinician and patient interviews, they were further refined into a list of seventeen concepts, each of which is detailed hereafter:

#### Convenience related to treatment

Five issues of concern related to treatment convenience were discussed: using tablets, receiving injections, performing self-injections, requiring long-term treatment and effects on daily activities. Tablets were seen as rapid and easy to use, carry and swallow. However, timings, dose complexity, dose variability and compliance when traveling were major constraints. For injections, some patients reported being not bothered by or afraid of the procedure. However, injections were also seen as unpleasant, painful, limiting on travel and time, a source of anxiety and fear, associated with allergies and subject to dose variability. Some patients expressed a willingness to perform self-injections, preferring the independence and the fact that regular blood tests are not required. Others spoke of fear and difficulties in performing the injection, obtaining the products, transporting the equipment and problems associated with age. A few patients felt that they would become used to long-term treatment but required regularity and organization. Daily activities that were reported to be affected by treatment included changes in sports, leisure, travel, work, gardening and the amount of injuries incurred.

#### Convenience related to blood monitoring

Some patients and clinicians reported that regular blood tests could interfere with daily life and work and represented a constant reminder of their disease condition. The frequency of tests, social stigma, compliance, costs, transport requirements, time spent waiting and pain associated with the tests were also issues. Advantages included feelings of confidence and reassurance and a means for checking treatment efficacy.

#### Perceived efficacy

Issues of concern included relief of symptoms, confidence in the treatment and protection against future thromboembolic events. Some patients felt more confident with injections than oral treatment. Other patients preferred oral treatment and some reported no perception of treatment efficacy.

#### Perceived safety – Side effects

Some patients and clinicians suggested that no concerns were apparent regarding side effects, treatment interactions or food interactions. However, local bruises, discomfort and gum bleeding, social and physical stigmas, allergies, hair loss and memory loss were sometimes reported as important side effects. Fears associated with bleeding and negative interactions with other treatments were also a concern. A few patients expressed that the need to be more cautious with food choice, alternative treatments and physical activity were constraining.

#### Patient preference on the type of administration route

Patients' preferences varied and included preferences for oral treatment, self-injection and injection by a third party.

#### Autonomy

Some patients perceived an improvement in their autonomy, whereas others did not yet report a gain. Some patients reported better autonomy with oral treatment and self-injection than injections performed by a third party. However, other patients expressed that blood tests and clinician follow-up lower anxiety and provide a sense of confidence. Feelings of dependency on the treatment were noted. The importance of compliance was sometimes expressed by both clinicians and patients. Several patients felt that compliance and monitoring were better with injections, and easier when not performed at home. Compliance was also seen as being related to age, the frequency of treatment intakes and the amount of side effects experienced.

#### Medical follow-up

Issues of concern included feelings of confidence and satisfaction with medical staff availability, reassurance and care received. Performing blood tests and good communication were important for feelings of confidence. Follow-up visits were sometimes seen as a constraint.

#### Information provided to patients by clinicians (clinicians' point of view)

As described by clinicians, information included explanations on the disease and its origin, treatment requirements, mode of action and side effects, as well as information on blood tests, emergency procedures and interactions with food. A few clinicians stated that the level of information was low, particularly with regard to vital prognoses and that comprehension was also low for older patients.

#### Information provided to patients by clinicians (patients' point of view)

As described by patients, information was similar to what clinicians described. However, some patients specified that more information was needed regarding disease background, sequelae, conditions of treatment use, duration of use, side effects, emergency procedures, food and other treatment complications. Some patients were concerned with understanding the variance in blood rates and forgetting the information provided. Some patients preferred to have information while others did not.

#### Patients' expectations

Expectations included being cured, symptom relief, prevention of future events, having no complications or side effects, a decrease in health risk and treatment efficacy. Some patients expected short-term treatments and limited duration of disease, others expected not to have immediate results. Some patients expected having injections, that the treatment would be easy-to-use and that they would have medical support and follow-ups. Some patients had no specific expectations.

#### Wishes

Some patients' desires included having information on the disease risk and origin, on treatment and its interactions with food and other treatments, and seeing blood test results. Patients' opinions varied with regard to whether they wanted blood tests or not. Some patients requested symptom relief, a simplified regular dosage, a once a week injection and more exposure to medical staff.

#### Worries and anxiety

These feelings were reported by some patients to be related to the disease (heredity, chronology, complications, symptoms), the treatment (side effects, drug interactions, hospital visits, injections, blood test results, forgetting the treatment), stopping the treatment (fear of relapse), mortality, or changes that might occur in work, the future and during pregnancy.

#### Perception of disease and symptoms

A few patients were concerned with issues related to the disease and symptoms, including swelling in the leg and arm, stiffness of the leg, pain in the leg, chest, arm and back, shortness of breath, dyspnea, heart palpitations, nausea, vomiting, vertigo, dizziness, headache, fatigue, tiredness, coughing, choking (blood in particular), metabolism problems, heavy perspiration, high cholesterol, bad feelings, cold fingertips and toes, and fever.

#### Symptom alleviation

According to patients, symptom alleviation could include treatment, rest, appropriate clothing, proper positioning and cold water.

#### Impact of disease on physical activities

For some patients, physical impact included limitations in activities such as walking, sports, going out, vacationing and gardening. Patients also reported avoidance of some movements and shortness of breath.

#### Psychological impact of the disease on patients

Patients sometimes described the disease as affecting their mood and general awareness.

#### Impact of disease on daily life

Such impact included effects on the patients' sleep, aesthetic appearance, dealing with local stigmas, lower energy levels, wearing compression stockings and changes in daily activities to avoid injuries and becoming too tired.

Concepts were organised into a list of four domains ("Treatment Expectations", "Convenience", "Anticoagulant Treatment Satisfaction" and "Burden of Disease and Treatment"), and then prioritised according to their relevance in assessing treatment satisfaction and convenience as well as on their ability to distinguish between different types of treatment (Table 3). Thirty detailed concepts corresponding to the previous described concepts were established, each being evaluated for validity across countries and disease conditions. One detailed concept on cost and one on the overall satisfaction with anticoagulant treatment were included in the short list. In contrast, no items were developed for the concept 'information about disease and anticoagulant treatment' as this concept was considered unessential to treatment assessment.

**Table 3:** Short list of concept classification provided from patient interviews

**Domains**	**Concepts**	**Detailed concepts**	**Matching item in final PACT-Q**
**Treatment Expectations**	Efficacy	Reassurance; occurrence or recurrence of events	#A1
		
		Alleviation of symptoms (e.g. pain)	#A2
		
		Recovery	None
	
	Safety	Safe administration (mistakes in administration)	#A5
		
		Minimisation of side effects (bruising, bleeding)	#A3
	
	Convenience	Easy administration/route	#A4
	
	Autonomy	Keeping control (of schedule, disease, treatment)	# A6
	
	Cost	Cost	#A7

**Convenience (evaluation)**	Treatment	Administration/route	#B1
		
		Bothersomeness, constraints	#B2
		
		Dose adaptation	#B3
		
		Drug-drug interactions	#B4
		
		Drug-food interactions	#B5
		
		Flexibility (storage, handling, place, context)	#B6
		
		Time (planning, time spent, transport)	#B7
	
	Blood test procedure	Constraints (frequency of monitoring)	None
		
		Time (planning, time spent, transport); trip	#B8
		
		Bothersomeness, constraints	#B9
	
	Autonomy	Dependence on nurse, caregiver	#B10

**Anticoagulant Treatment Satisfaction (evaluation)**	Efficacy	Reassurance, occurrence, or reoccurrence of events	#D1
		
		Alleviation of disease symptoms	#D2
	
	Safety	Side effects	#D3
		
		Safe administration (mistakes in administration)	None
	
	Autonomy	Route of administration	None
		
		Keeping control of disease/worries about not keeping control	#D4
		
		Satisfaction with staff	#D5
		
		Preference with treatment form	#D6
		
		Overall treatment satisfaction	#D7

**Burden of Disease & Treatment (evaluation)**	Impact of side effects, disease symptoms and blood monitoring	On daily activities	#C1
		
		On work	#C1
	
	Discomfort	Because of bruising, pain	#C2
	
	Treatment interruption	Worries about interrupting treatment	#B11

### Item generation

**Figure 1:**
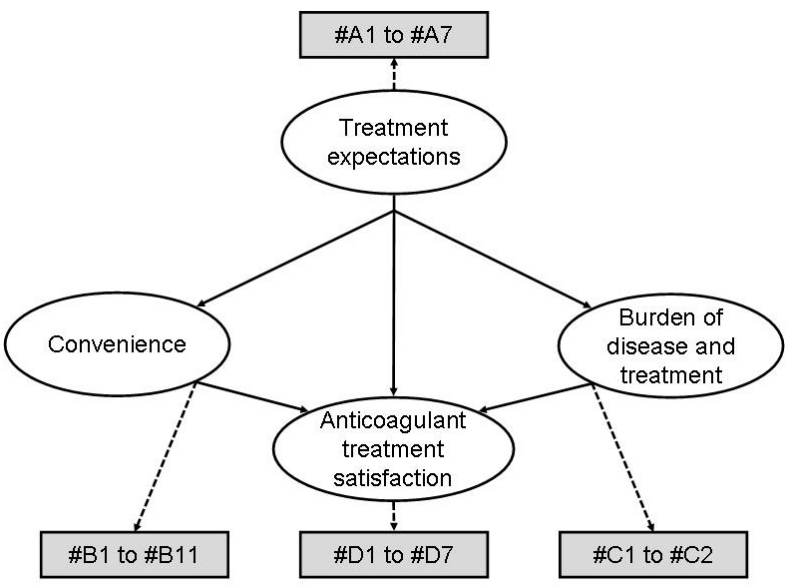
**Refined conceptual model of the PACT-Q pilot version**.

Using the detailed concepts listed and based on patients' verbatim transcripts, three language versions (French, Dutch and US English) of the pilot questionnaire were created and validated, each containing 27 culturally equivalent items grouped into four domains: "Treatment Expectations" (7 items), "Convenience" (11 items), "Burden of Disease and Treatment" (2 items) and "Anticoagulant Treatment Satisfaction" (7 items) (Figure 1). No item was created for the detailed concepts about 'recovery', 'constraints (frequency of blood monitoring)', 'safe administration (mistakes in administration)', 'route of administration', and one single item was created for the concept 'impact of side effects, disease symptoms and blood monitoring on work and daily activities' since these detailed concepts were either covered elsewhere or qualified as a source of misunderstanding. Answers were designed according to 5-point Likert response scales. The questionnaire format was subsequently divided into two parts: the first part to measure expectations (7 items), to be administered before receiving treatment, and the second to measure convenience, burden and treatment satisfaction (20 items), to be administered after having received the treatment.

### Content validity testing

#### Description of patients

Nineteen patients were recruited in France, the Netherlands and the US, seven with DVT (the Netherlands, n = 2; US, n = 2; France, n = 3), five with PE (the Netherlands, n = 1; US, n = 2; France, n = 2), six with AF (n = 2 in each country). One patient who had not had a thromboembolic event but who had an increased risk of thrombosis due to major abdominal vein surgery was included due to prior experience with self-injection. Patients' socio-demographic and clinical characteristics were similar to those interviewed for the concept development. Among the 19 patients interviewed, nine were males, ten were active, seven were retired, one was a housewife, and one was not working due to present health status. The mean age of patients was 52.3 years, the mean disease duration was 2.8 years. All patients were receiving anticoagulant treatment. Three patients out of 19 were receiving injections of anticoagulants and seven patients had had an experience with self-injection of anticoagulants. Twelve patients were to receive a lifetime treatment.

#### Patients' comments

In the three countries, patients found the questionnaire clear and easy to understand in general. They found the length appropriate and the layout was well accepted. Patients gave specific minor comments on each item of the questionnaire that were incorporated. These corresponded to re-wording or to adding more detail to the questions to make them more accurate. No item was deleted or added after pilot testing. Final wording of the questionnaire was decided and validated by the advisory board. The questionnaire was named the PACT-Q (Perception of Anticoagulant Treatment Questionnaire). The first part was labeled PACT-Q1, and aimed at measuring expectations. The second part was labeled PACT-Q2 and aimed at measuring convenience, burden of disease and treatment, and anticoagulant treatment satisfaction.

### Linguistic validation

Linguistic validation was performed on the PACT-Q into eleven additional languages (Australian English, Belgian Dutch, Belgian French, Canadian English, Canadian French, Czech, Danish, German, Italian, Polish and US Spanish) to obtain conceptual equivalence between the target language versions and the original questionnaire.

#### Conceptual and linguistic issues

As sometimes no direct word equivalent exists in a target language (either linguistically or culturally), an appropriate translation was put forward, discussed with the developer and implemented in the translations when they were found acceptable. The aim was to retain options considered simple, colloquial and conceptually equivalent to the original. For example, "bother" as a verb could encompass a range of feelings on the part of the respondent. In the languages where this alternative was possible, it was considered preferable to use an equivalent of "to bother" instead of an equivalent of "to annoy/to worry", as these convey a slightly different meaning. In the languages, when more idiomatic, possible alternatives used for "how bothered are you by... " were similar to" how much are you preoccupied by", "do you feel it is a burden to" or "how inconvenient is it for you". Overall, terminological differences did not have an impact on the interpretation of the question.

Other examples of conceptual or linguistic issues were as follows: in some cases, the equivalent in the different languages of "dependent on others" was confused with a concept of addiction or of embarrassment. In addition, it was felt a little degrading, as though it were a crippling disease, psychologically speaking. The equivalent of "to have more need of others' help" was therefore used, as being more understandable and culturally acceptable.

In some languages, the "follow-up" concept was difficult to express. "Follow-up" of treatment included assessing the status of the disease. In addition, the word used to render "follow-up" referred to subsequent examinations, e.g. visits to the doctor (for check-ups). It referred to the patient and not really to the disease. Literal equivalents of "follow-up" also appeared to be technical terms used by clinicians rather than patients, who viewed them as being complementary to the medicine used in their treatment. In languages for which "follow-up" proved a difficult term to translate or understand, alternative expressions such as "monitoring" were found acceptable.

#### Pilot testing

In each country, the respective PACT-Q version was tested on five patients with either DVT, PE or AF and following an anticoagulant treatment. The mean age of the respondents was 57 years across countries, ranging from 47 to 64 years. Out of the 55 respondents interviewed, 27 were men. Respondents took an average of 12 minutes to complete the questionnaire (ranging from 5 to 20 minutes across countries).

Overall, the questionnaire was found to be clear, relevant and appropriate to the circumstances. The examples provided were perceived as very helpful and the questions were therefore well understood. Some respondents found the questionnaire to be complete and short. Others had minor comments including redundancy or similarity for some questions.

The items relating to interaction of other drugs and food with the anticoagulant treatment caused a certain level of worry and anxiety. However, the difficulties expressed by respondents did not concern the wording of the question but rather the desire for more explanations or information. In the "Convenience" domain, the question "How worried are you about having to interrupt or stop your anticoagulant treatment?" seemed to confuse certain respondents, because the possibility of having to interrupt or stop their treatment had not occurred to them before the question suggested it. Respondents could not imagine why they would stop treatment unless they had recovered from their illness.

After further analysis of their comments, it appeared that the respondents understood the question, but that as they had never considered the question, it raised many new questions for them. Thus, the items were kept. Please note here that the items with their specific wording are available on request from the authors.

## Discussion

Numerous studies have shown the relationship between patients' satisfaction and treatment compliance, which may lead to treatment failure [20-22,33]. Reported outcomes on patients' satisfaction are therefore of interest when one wants to measure treatment benefits, to understand the needs and expectations of patients and to explain treatment compliance. Satisfaction "instruments" can provide direct comparison of treatment administration routes and procedures; they present succinct evaluations of patients' perceptions through the use of a short set of simple questions; they offer additional relevant information that cannot be assessed through clinical endpoints; they can be applied to clinical studies with fixed timelines.

Previous attempts at measuring satisfaction with anticoagulant treatment have been published [7,23-26,29]. In a few cases, patient satisfaction questionnaires were developed with a focus on the structures and processes of anticoagulant medical care. Although some information can be derived from the medical care-satisfaction literature, studies are often too broadly focused to be of value in the assessment of satisfaction with specific regimens or therapies, which is particularly noticeable when questionnaires attempt to measure both satisfaction and HRQoL in one instrument [34]. Indeed, while the conceptual model underlying satisfaction suggests that it should be measured against expectations [35], HRQoL is by definition related to a specific condition or disease and is a multidimensional construct requiring the measurement of physical functioning, mental functioning, social functioning and emotional well-being [36,37].

The PACT-Q was developed as a specific treatment satisfaction instrument for thromboembolic patients with anticoagulant treatment.

### Development of PACT-Q

The development of the PACT-Q followed a rigorous qualitative, international multi-step approach, involving a literature review, simultaneous patient and clinician interviews in three languages, advisory board validations, pilot-testing of the preliminary instrument in the same three countries and linguistic validation into eleven language versions. Patient and clinician interviews identified a range of views regarding the nature and management of treatment and of disease. Comprehensive review of all decision processes and materials by experts, questionnaire specialists and linguists at each appropriate stage of the development process ensured the thorough development and validation of the questionnaire and allowed for its immediate linguistic adaptations. The resulting PACT-Q is a brief, easy-to-use, high quality patient-completed questionnaire, available in 14 different languages.

### Number of interviews necessary to ensure the relevance and comprehensiveness of the instrument

The rationale for setting the number of interviews in qualitative research should in theory be based on the concept of saturation [38]. Roughly, saturation is achieved when additional interviews no longer add new concepts to the list. However, in our simultaneous development design, the evidence of the comprehensiveness of the identified concepts was the concordance in findings from the three different countries, rather than the concordance between consecutive sets of interviews in one single country.

### Multinational applicability of PACT-Q

A well-identified limitation to generalisability of PRO instruments is due to the cross-cultural differences. The PACT-Q was developed simultaneously in multiple languages, i.e. French, US English and Dutch, and underwent rigorous linguistic validation. This reduces the risk of systematic measurement error at the item level (i.e. item bias) and ensures the consistency of concepts across different cultures. The procedure was intended to yield 'optimal' measures for adaptation into different cultures. Additionally, it aimed to produce a measure that was less susceptible to cultural differences than a questionnaire developed only in one language and followed by translation into other languages [39].

Linguistic validation of the PACT-Q into Australian English, Belgian Dutch, Belgian French, Canadian English, Canadian French, Czech, Danish, German, Italian, Polish and US Spanish was established according to a rigorous development and translation process to ensure conceptual equivalence and cultural relevance across all languages [31,32]. To achieve this, a comprehensive review of the concept short list and the conceptual definitions of the original items was carried out. To ensure understanding of the underlying concepts of the PACT-Q, and hence conceptual equivalence across all countries, the list of concepts was systematically compared across all countries. Such conceptual transparency adheres to the current guidelines on thorough questionnaire development and linguistic validation, and ultimately allows for the international comparison and pooling of data to generate easily interpretable summary scores [40]. The availability of the PACT-Q in 14 language versions makes it internationally applicable.

The simultaneous development conducted in three European countries actually facilitated the linguistic validation of the questionnaire in eleven other languages in western countries. However, an assessment of cross-cultural validity will be a necessary step in regions where the experience, values and priorities of the patients may differ.

### Satisfaction and expectations

The division of the PACT-Q into PACT-Q1 (expectations) on the one hand and PACT-Q2 (convenience, burden and treatment satisfaction) on the other hand for prior and follow-up assessment, is expected to facilitate the interpretation of the future scores. As several authors agree, satisfaction is at least partly linked to prior expectations [13,15]. In therapeutic trials, the assessment of expectations could be useful to 1) describe the level of expectations for several treatment attributes at baseline, and 2) be part of multivariate models explaining the respective impact of treatment characteristics and patients' expectations on the levels of satisfaction reported by the patients.

However, as expectations result from former experience and information received, the contents of a questionnaire are limited by the range of experience the patients interviewed have had, as well as by the information they have received. It is likely that changes in the disease as well as in future new therapies (for example curative treatments) might impact the nature and the levels of expectations as well as those of satisfaction.

### Next steps in the validation process

The heterogeneity of the target population at each stage of the development and validation process ensured that the relevance of the PACT-Q extends to a variety of anticoagulant patients with different condition severities, socio-demographic characteristics and treatment formulations. As AF, DVT and PE patients account for a large proportion of anticoagulant patients, the PACT-Q is expected to be used for assessment of the issues of greatest concern for a wide spectrum of patients.

The qualitative research process used to develop the PACT-Q aims at providing researchers with an instrument based on strong assumptions regarding its content and its structure. However, validation is an ongoing process, and the true worth of an instrument only becomes clear with its use. Further quantitative psychometric validation steps will be necessary to consolidate its validity and suitability for application in clinical research studies.

## Conclusion

As an increasing number of safer and equally effective anticoagulant treatment alternatives become available, satisfaction will be an important variable to assist in product differentiation. The PACT-Q, a rigorously developed treatment satisfaction questionnaire, is able to assess satisfaction pertaining to different types of anticoagulant treatments and is now available in 14 languages for use with AF, PE and DVT patients. Its structure and psychometric properties were validated and are presented in a separate manuscript [Prins MH, Guillemin I, et al: Scoring and psychometric validation of the Perception of Anticoagulant Treatment Questionnaire (PACT-Q), *Unpublished*]

## Competing interests

The work was funded by Sanofi-Aventis, Research and Development. AM and BA are paid consultants to Sanofi-Aventis, Research and Development. PC is an employee of Sanofi-Aventis, Research and Development. The other authors have no conflict of interest.

## Authors' contributions

All authors provided intellectual contributions to this manuscript. Clinical directives given by MP included defining the questionnaire and study objectives, making final decisions and data interpretation. Methodological directives provided by BA included conception and design input for questionnaire development and data interpretation. Other advisory board members validated decisions and contributed to data interpretation. AM was responsible for data acquisition, analysis and interpretation. PC provided input on the questionnaire development and data interpretation.
